# Information about others’ choices selectively alters risk tolerance and medial prefrontal cortex activation across adolescence and young adulthood

**DOI:** 10.1016/j.dcn.2021.101039

**Published:** 2021-11-18

**Authors:** Barbara R. Braams, Juliet Y. Davidow, Leah H. Somerville

**Affiliations:** a Department of Psychology and Center for Brain Science, Harvard University, Cambridge, MA, USA; b Department of Clinical, Neuro, and Developmental Psychology, Faculty of Behavioral and Movement Sciences, Vrije Universiteit, Amsterdam, The Netherlands; c Department of Psychology, Northeastern University, Boston, MA, USA

**Keywords:** Risk, Ambiguity, FMRI, Adolescence, MPFC

## Abstract

Adolescence is associated with major changes in the cognitive, emotional and social domains. One domain in which these processes intersect is decision-making. Previous research has shown that individuals’ attitudes towards risk and ambiguity shape their decision-making, and information about others’ choices can influence individuals’ decisions. However, it is currently unknown how information about others’ choices influences risk and ambiguity attitudes separately, and the degree to which others’ choices shape decision-making differentially across development from adolescence to young adulthood. The current study used a computational modeling framework to test how information about others’ choices influences these attitudes. Participants, aged 14–22 years, made a series of risky and ambiguous choices while undergoing fMRI scanning. On some trials, they viewed risky or safe choices of others. Results showed that participants aligned their choices toward the choice preferences of others. Moreover, the tendency to align choices was expressed in changes in risk attitude, but not ambiguity attitude. The change in risk attitude was positively related to neural activation in the medial prefrontal cortex. Results did not show age related differences in behavior and corresponding neural activation, indicating that the manner in which adolescents are influenced by peers is not ubiquitous but rather, is highly context-dependent.

## 1 Introduction

Many important decisions in life have uncertain outcomes, such as decisions in the financial, health and social domains. Although the outcomes associated with these choices are uncertain, they can have significant consequences. Given the importance of decision making strategies on life outcomes, it is important to understand what factors contribute to decision making and the role of contextual factors in decision making. Two important factors for decision making under uncertainty are attitudes towards risk and ambiguity. Previous work has shown that information about others’ choices has a robust influence on decision making. Seeing information about others’ previous choices makes one more likely to also select that option, an effect seen in the more safe (Braams et al., 2019) as well as the more risky direction (Chung et al., 2015). In this study, we investigate how seeing information about others’ previous choices specifically affects attitudes towards risk and ambiguity. We use functional magnetic resonance imaging (fMRI) to investigate which neural processes underlie changes in these factors of decision making when individuals have access to information representing the previous choices made by others. To evaluate whether adolescents are particularly attuned to this information, we tested a developmental sample of participants between the ages of 14 and 22 years. Each of these components is discussed in detail below.

## 1.1 Risk and ambiguity

Experimental studies provide a unique opportunity to decompose complex processes such as decision making into components to study how each of these factors selectively influences decision making. Two important factors for decision making are attitudes towards risk (i.e., choices with variable outcomes and known probabilities of these outcomes), and ambiguity (i.e., choices with variable outcomes with unknown probabilities of outcome). Attitudes towards risk and ambiguity can be dissociated with a widely used computational modeling approach (Blankenstein et al., 2016, Levy et al., 2010, Tversky and Kahneman, 1992, Tymula et al., 2012). In general, individuals show both risk aversion (Tversky and Kahneman, 1992) and ambiguity aversion (Blankenstein et al., 2016, Ellsberg, 1961, Tymula et al., 2012). Attitudes towards risk and ambiguity show weak relations, indicating that they capture different elements of decision making under uncertainty (Von et al., 2011).

FMRI research provides information on how human brain functioning supports decision making processes. Experimental studies have identified neural regions involved in general decision making, and more specifically permits identification of neural responses that scale with the riskiness and ambiguity of the choice options. Although the exact neural mechanisms tracking risk and ambiguity during decision making is not fully understood, previous work and a recent meta-analysis have shown that both risky and ambiguous decision making engage neural regions related to salience (i.e. insula and anterior cingulate cortex), valuation (i.e., striatum), and executive control (i.e. parietal and prefrontal regions) (Blankenstein et al., 2017, Huettel et al., 2006, Krain et al., 2006, Levy et al., 2010, Poudel et al., 2020). These findings suggest that evaluating risk and ambiguity may require recruitment of the brain’s distributed affective and cognitive systems.

Ultimately, choices are made based on an integrated value signal representing each of the choice options, known as subjective value (Tversky and Kahneman, 1979). The subjective value of choice options is constructed of different parts: the objective characteristics of the choice options (i.e., riskiness and ambiguity), and the weight that subjects attribute to the riskiness and ambiguity of these options (i.e., risk and ambiguity tolerance) (Levy et al., 2010, Tversky and Kahneman, 1992, van den Bos et al., 2018). The same choice option can therefore have a different subjective value for individuals based on the risk and ambiguity tolerance of the individual. The subjective valuation of different choice options is encoded in the ventromedial prefrontal cortex (Bartra et al., 2013, Clithero and Rangel, 2014, Kable and Glimcher, 2007, Levy et al., 2010).

### 1.2 Social context

Besides risk and ambiguity, the social context is another factor that is highly relevant for decision making and has been shown to influence decision making under uncertainty. A large body of literature shows that people tend to conform to others’ choices and behavior (Brechwald and Prinstein, 2011, Cialdini and Goldstein, 2004, Zaki et al., 2016, Zaki et al., 2011). There are many different ways in which others can influence decisions, ranging from active involvement of others in the decision making process to passive observation. In the current study, we focus on one specific type of social context: how information about others’ previous choices influences decision making.

A real-life example is when faced with the decision to drive home after drinking or to arrange alternative transport. Knowledge about the previous decisions of others in a similar situation can influence whether we judge it acceptable to drive. Prior experimental studies have shown that people are prone to follow previous decisions of others (Albert et al., 2013, Blankenstein et al., 2016, Braams et al., 2019, van Hoorn et al., 2016). Adults make more risky decisions when they see that someone else makes risky choices, and conversely make less risky decisions when they see that someone else prefers the safe option (Chung et al., 2015). In this study, computational models indicated that information about others’ choices alters the subjective value of the choice option selected by the peer. The increase in subjective value was related to activation in the ventromedial prefrontal cortex. It is currently unknown how information about others’ choices separately influences risk and ambiguity attitudes and its related neural correlates.

### 1.3 Age related changes in decision making

Adolescence is the stage in life between childhood and adulthood that begins around the onset of physical puberty and ends with the assumption of independent roles. Adolescence is an important period in life associated with both changes in decision making under uncertainty and changes in the social domain. As children enter adolescence, peers become more important and adolescents show heightened risk taking behavior in the real world when they are with their peers (Doherty et al., 1998, Eaton et al., 2010). Because of the highly salient social context and the changes in risk taking behavior during adolescence, this age group can give unique insight into the process of decision making under uncertainty. Two behavioral studies tested the influence of information about previous choices of supposed peers on risk and ambiguity attitudes. One study tested how previous risky and safe choices of others influenced participants’ choices on risky and ambiguous choice options. In this study, late adolescents, compared to children, early adolescents and adults, were more likely to follow peers’ choices for a safe option and less likely to follow a risky option (Braams et al., 2019). There was no evidence for an interaction between social context and riskiness or ambiguity of the choice options. However, this study did not use a computational framework to derive risk and ambiguity tolerance parameters for each individual, which could provide a more precise index of behavior on which to evaluate the influence of peers’ choices. For this reason, the present study incorporates a computational framework.

A second study utilized a computational modeling approach to disentangle the effects of risky choices of others on risk and ambiguity tolerance (Blankenstein et al., 2016). In this study, participants aged 10–25 years made decisions with varying levels of risk. In one condition they did not see information about others’ choices, and in another condition they were presented with previous choices of another individual. This study found that participants increased their selection of risky choice options when presented with the previous risky choices of another person. The change in choice behavior was related to an increase in risk tolerance, and no changes in ambiguity attitude were observed. The change in risk tolerance was highest for the younger participants in the study and linearly decreased into young adulthood. However, this study did not test the influence of safe choices of a peer. Together, these studies show that adolescents incorporate information about previous choices of others when making decisions. The current study extends this work by investigating how these safe and risky choices change risk and ambiguity tolerance across age.

### 1.4 The current study

The current study evaluated which neural mechanisms underlie changes in risky and ambiguous decision making when participants are informed about previous safe and risky choices of others, and whether these processes change with age from adolescence to young adulthood. Participants made a series of choices between a sure, safe, option and a risky option with variable outcomes. The risky option varied in level of risk (i.e., outcome variability), and ambiguity (i.e., uncertainty about the chances of winning). To test the effects of social information, for some decisions, participants received information on choices of supposed previous participants in the study. These supposed other participants reflected a generally risk-tolerant or risk-averse choice strategy. As a comparison condition, participants made choices without information about others’ previous choices. Participants’ neural responses were recorded while making decisions.

We utilized a computational modeling approach to estimate parameters reflecting each participant’s risk and ambiguity attitudes. We hypothesize that risk tolerance, as estimated by parameters in the computational utility model, will increase when participants are observing risky choices made by peers. Conversely, we expect that risk tolerance will decrease when seeing safe choices of others. It has been suggested that following safe choices of others might be a particularly salient context for social learning, as safety cues from others can facilitate avoidance of negative outcomes (Ciranka and van den Bos, 2021).

We expected that adolescents would show a distinct pattern of decisions when information about others’ choices is present. However, different predictions could be derived from different literatures on adolescent social learning and decision making. One line of prior work has demonstrated that relative to adults, adolescents are especially prone to learning from direct experience rather than following the guidance or instructions by others (Decker et al., 2015). This may lead to a prediction that adolescents are especially motivated to make choices that are independent of the views of others, which would lead them to be less likely than adults to shift risky choice behavior based on the previous choices of peers. A second line of work emphasizes adolescents’ propensity to be influenced by peers. Previous work has demonstrated that direct observation by peers will induce riskier choices by adolescents more so than adults (Albert et al., 2013, Chein et al., 2011), which might lead to an opposite prediction – that adolescents would be more influenced by peers’ choices. Finally, recent perspectives have emphasized the potential for an asymmetric effect of peers on risky relative to safe choices during adolescence, which aligns with prior empirical findings (Braams et al., 2019, Chung et al., 2020, Engelmann et al., 2012). This perspective emphasizes that information about *safe* choices of peers could exert greater impact than information about risky choices during adolescence, as observing others behave safely during exploration is a particularly important signal that would facilitate avoidance of negative outcomes when encountering new environments or information.

These previous studies and perspectives do not yield a highly cohesive set of behavioral predictions; therefore, we did not make strong behavioral hypotheses for the present study. Rather, the objective of the present study was to generate robust data to help resolve these competing perspectives. In the brain, we predicted that changes in risk tolerance would show a positive relationship with activation in neural regions previously related to risk processing and integration of different sources of information including the ventral striatum, insula and ventromedial prefrontal cortex (Blankenstein et al., 2017, Blankenstein et al., 2018, Levy, 2017). In parallel with potential age-related differences in the computational model estimates of risk and ambiguity attitudes, we expected that patterns of neural activation would track with age-related changes in risk and ambiguity tolerance in the social information conditions. Together, these analyses provide more insight into the underlying processes that give rise to changes in risky and ambiguous decision making in a social context*.*

## 2 Methods

### 2.1 Participants

A total of 69 participants between 14 and 22 years old participated in the study. The selection of the age range for the current study resulted from a balance between practical concerns regarding feasibility of the data collection and inclusion of the age range of interest. The results of our previous study (Braams et al., 2019) showed that the effects of observing peer choices were strongest in older adolescent group, age range ~16–18. By reducing the age range, we increased the sample density for the ages selected in the sample, allowing a focus on the age range of interest. The participants in the current study comprised an independent sample with no overlap in participants tested in our previous work (Braams et al., 2019).

Individuals were excluded from participation if they endorsed a history of neurological disorders, head trauma, recent diagnosis of any psychiatric disorder, insufficient command of the English language to understand task instructions, or MRI contraindications. In the case of minors, parents were asked to report on these exclusion criteria. Exclusion criteria for data analysis for fMRI data included a motion cutoff of > 5 mm framewise displacement or > 15% framewise motion of more than 0.9 mm in two or more runs (Davidow et al., 2019, Siegel et al., 2014). Signal to noise ratio (SNR) was calculated as the mean/standard deviation averaged for each slice and then averaged across all slices. Any participant with two or more runs with excessive motion or signal to noise ratio values < 99 were excluded from data analysis. Additionally, participants were excluded if they did not finish data collection or if they did not believe the social manipulation. In total, four participants were excluded from final data analysis: one for incomplete data, one for low SNR, one for excessive motion, and one who did not believe the social manipulation (see task design). The average absolute motion across the three runs in the included sample was 0.48 mm (min=0.12 mm, max=2.46 mm). The total number of usable datasets was 65 (32 females), M_age_= 18.54, SD_age_= 2.61, Range_age_= 14.21–22.83. A chi-square test indicated that the proportion of males and females did not vary significantly across age (χ^2^(58) = 61.00, *p* = .37).

### 2.2 A priori power analysis

We performed a power analysis before beginning the current study to determine the sample size using the package simr (Green and MacLeod, 2016). Because we previously published a behavioral study using a similar paradigm with 99 participants aged 12–22 years old (Braams et al., 2019) we leveraged a subset of this prior independent dataset to calculate observed power for the behavioral effects of central interest.

In the current study, we focused on participants ages 14–22 years old. Therefore, for the observed power analysis we only included individuals of this age range, resulting in N = 65, after ensuring an equal distribution across age and gender. Note that participants were selected based on age and gender and not on behavioral performance in the task. As we were interested in age-related effects in the social conditions, we used the effect size of the Age x Social Risky and Age x Social Safe interaction effects on risky choices from the previous study. A sample size of 65 participants resulted in 96% power for the Age x Social Risky effect and 100% power for the Age x Social Safe effect. The power analysis indicated that a sample of 65 participants would provide sufficient power to detect age-related effects of the social vs solo conditions on risky choices at the behavioral level. The previous behavioral study did not employ computational modeling or brain imaging approaches. Thus, this power analysis cannot inform power for the computational models or the fMRI analyses.

### 2.3 Session

The total duration of the session was 3 h. Participants received $60 for participation and they could earn up to $23 in bonus money based on their choices in the task. Prior to participation, adult participants provided informed consent, and parent permission and participant assent were obtained for minors. This study was approved by the Committee on the Use of Human Subjects at Harvard University.

### 2.4 Task

#### 2.4.1 Trial Structure

**Fig. 1:**
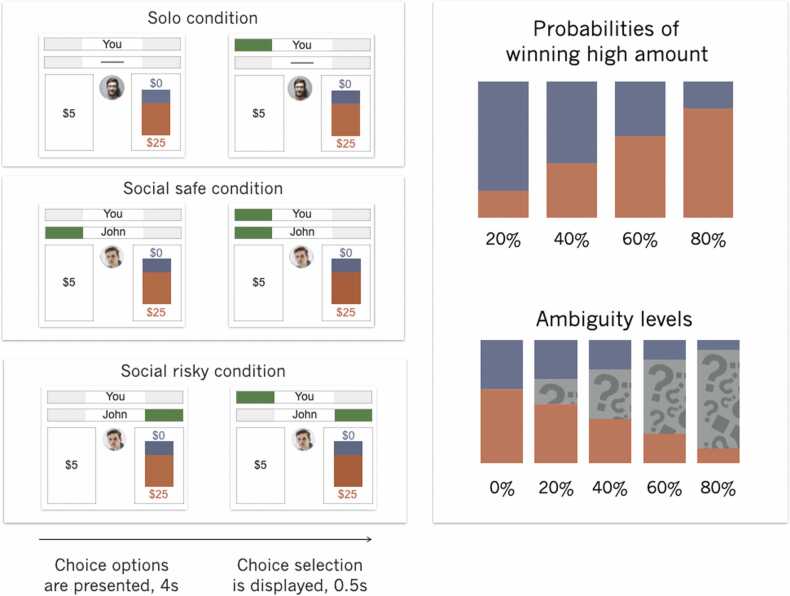
Task Design. Trials started with a screen showing the choice options (see panel A). One option, the safe option, was always a sure $5. The other option, the risky option, was a chance of winning zero dollars or a high amount of money varying between $3 and $76. Chances of winning the high amount of money (in red) varied between 20% and 80% (see panel B). For the ambiguous choices, part of the bar was occluded thereby introducing uncertainty about the chances of winning. Ambiguity levels varied between 0%−80% (see panel C). The example in the figure reflects all ambiguity levels for a trial in which the chance of winning the high amount (shown in red) was 60%. In the solo condition, participants made choices without availability of additional information about a peer’s choice. In the social conditions, participants saw a picture of a peer and their choice, displayed as a green bar over the selection. Trials in the social safe condition were defined as all trials on which peer selected the safe option, whereas trials in the social risky condition were defined as trials in which the peer selected the risky option. Peers were the same gender and age category as the participants. The example displays an 18–22-year-old male. After the trial onset, participants indicated their choice within 4 s, their choice was displayed for 0.5 s and trials ended with a variable jitter.

On each trial, participants made a choice between a safe option (a sure amount of $5) and a risky option (a lottery). For the risky option, there was a low and a high magnitude outcome. The low outcome of each lottery was always $0 and the high magnitude outcome varied between $3 and $76. The chance of winning the high magnitude outcome varied between 20% and 80% in increments of 20%. The expected value of the risky option (i.e., magnitude of the high amount multiplied by the odds of winning the high magnitude) varied between $1 and $60. The expected value of the safe option was always $5. To account for the fact that overall, people tend to be risk averse and to ensure sufficient variation in choices, the expected value of the risky option was higher than the expected value for the safe option on approximately 70% of the trials. Lotteries were represented graphically as bars with the colors of the bar representing the chance of winning different magnitudes (see Fig. 1).

Ambiguity was introduced into the lotteries by covering part of the bar. Ambiguity levels varied between 0% and 80%, in increments of 20%. Bars were covered proportionally, meaning that for a lottery with 20% chance of winning the low amount of money and 80% chance of winning the high amount of money and an ambiguity level of 60%, the total visible bar for the chance of winning the low amount of money was 8% and the total visible bar for the chance of winning the high amount of money was 32%. Consequentially, the optimal strategy (i.e., to ignore ambiguity) was the same for all levels of ambiguity. Any changes in decision making strategy for different ambiguity levels can therefore be attributed to the ambiguity level of the lottery. Note that participants were not informed of the optimal strategy and did not receive any additional information on the underlying probabilities.

We used a computational modeling framework to derive risk tolerance and ambiguity aversion estimates for each participant (also see ‘computational modeling’). To optimize model performance, the final set of presented trials was selected based on simulations of choice data and subsequent recovery of parameters of the computational model. Choice data were simulated for a range of parameter values for risk tolerance, rho 0.7 to 1.3 in increments of 0.3, and ambiguity aversion, beta ‐2 to 2 in increments of 1. Parameter recovery for the final data set was high. The correlation coefficient for rho and recovered rho was r = 0.94 and beta and recovered beta showed a correlation of r = 0.97.

On each trial, the two choice options (i.e., safe option and the lottery) were displayed and participants had 4 s to indicate their preferred choice, after which a confirmation of their choice was displayed for 0.5 s. Trials ended with a variable jitter between 1.21 s and 7.83 s, average jitter was 4.83 s (see Fig. 1). If participants responded within 4 s, the remaining time was added to the jitter. If participants did not respond within 4 s, the word ‘missed’ was shown on the screen for 1 s, the task then continued with the next trial. In total, eight different counterbalanced trial presentations were presented across participants. Probabilities of winning the high magnitude amount were presented either in blue or red, the location of the high and low magnitude amounts were presented on the top or bottom of the bar, and safe and risky choice options were presented on either the left or right side of the screen. Counterbalance assignment did not differ across age (χ^2^(56) = 48.40, *p* = .75).

### 2.4.2 Social manipulation

Participants were instructed that on some trials, they would see the choices of two peers of their choosing. On trials in which participants could see the choices of the peers, they were presented with a name and a picture of that supposed previous participant. Choices of peers were indicated by a green rectangle above their preferred choice (see Fig. 1).

Before the start of the task, participants rated 15 pictures of similar-aged and gender matched individuals. Participants rated these individuals on seven different questions assessing dimensions such as niceness, friendliness and popularity (see Braams et al., 2019 for details). Ratings were made on a continuous scale with anchors ‘not at all’ and ‘extremely’. Participants indicated their rating with a slider on the scale. Participants were told that these people were previous participants in the study and to make it believable that we took a picture of previous participants, the experimenter also took a picture of the participant. To ensure that participants saw choices of those individuals that they were most interested in, participants were asked to select two individuals for whom they would see choices in the task. Selected individuals received higher ratings on each of the seven different dimensions (i.e. attractiveness, possibility of becoming friends, niceness, popularity, similarity, whether the participant thought the other person was more attractive than they are and whether the participant thought the other person was more popular than they are, than non-chosen individuals; see Supplementary Fig. 1).

We randomly assigned the two selected peers to be the risky peer and the safe peer. The ‘Risky peer’ made 75% risky choices and 25% safe choices, whereas the ‘Safe peer’ made 75% safe choices and 25% risky choices. There were no statistical differences between the ratings for the safe and risky peer on any of the dimensions and participants’ choices were not related to the rating of the peers on any of the dimensions, see Supplementary Materials. Note that although the ‘peers’ each showed a distinct decision making profile (i.e., risky or safe), collapsing across the peers participants yielded 50% risky choices and 50% safe choices in aggregate. All ambiguity levels and all risk levels were distributed across trials for the peers, meaning that all ambiguity levels and all risk levels were shown in both conditions. See Supplementary Materials for details of selection of choices depicted in the social risky and social safe trials. Importantly, participants were never instructed to follow the choices of the peers.

We used a funnel debrief interview to assess whether participants believed the social manipulation. Participants were asked whether they paid attention to the choices of the other participants and if they answered that they did not, follow-up questions determined why the participant did not pay attention to the choices and whether the participant believed the manipulation. One participant indicated that they did not pay attention to the choices and that they did not believe the social manipulation; this participant was excluded from analyses. All other participants indicated that they believed the manipulation. Participants were fully debriefed about the social manipulation at the end of the session.

#### 2.4.2.1 Conditions

Trials displaying peer choices were presented intermixed with choices in which participants did not see any additional choice information (solo condition). Choices in the solo condition were used to quantify baseline risk preferences. In total, this resulted in three conditions: solo, social safe, and social risky. Choice simulation and subsequent parameter recovery (Wilson and Collins, 2019; see Supplementary Materials for details) showed that 60 trials per condition were needed for an accurate estimation of the risk and ambiguity tolerance parameters. The full time to perform the task for participants is 35 min, which we judged to be too lengthy for the developmental sample to complete inside of the MRI. Thus, participants performed part of the task inside the scanner and part of the task outside of the scanner. Trials presented during fMRI scanning included all levels of risk and ambiguity levels of 0%, 40% and 80%, for a total of 108 trials. Additional trials were presented outside of the scanner with all risk levels and with ambiguity levels 20% and 60%, adding 72 additional trials of behavioral data.

### 2.5 FMRI data acquisition

Data were collected on a 3 T Siemens Tim Trio scanner, with a 32 channel head coil. A high-resolution 3D T1-weighted anatomical image was collected using multiecho multiplanar rapidly acquired gradient-echo (MEMPRAGE) sequence (repetition time=2260 msec., echo time=1.69, 3.55, 5.41, 7.29 msec., flip angle=7°, field of view=256 mm, slice thickness=1 mm, voxel size=1x1x1 mm). Functional MRI data were acquired over three runs of 183 volumes each. Functional data were acquired with a T2 * -weighted EPI sequence with the following parameters: repetition time= 2 s, echo time= 31 msec., field of view= 206 mm, flip angle= 80°, voxel size= 2.4 × 2.4 × 2.4 mm, multi-band acceleration factor: 3, slice thickness: 2.4 mm, total number of slices per TR= 66.

## 2.6 Data analysis

### 2.6.1 Behavioral data analysis

#### 2.6.1.1 Raw choice data

All raw choice data were inspected for outliers, defined as |z|> 3. No outliers were identified for mean percentage risky choice for any of the conditions. To test whether the percent risky choices was different across conditions, we first calculated the mean percent choices for the risky option for the solo, social risky and social safe conditions. To test whether the social manipulation significantly altered percentage choices for the risky option, we performed *t*-tests to compare percentage choices for the risky option in each of the social conditions and the solo condition. In these analyses, we compared only those trials that were presented in both the solo condition and the social condition of interest to accurately reflect differences in risky choice based on the social information instead of the choice options that were presented.

#### 2.6.1.2 Computational modeling

We used a computational modeling framework to derive risk tolerance and ambiguity aversion for each participant. We used a well-established power utility function to model the expected utility of each option (Blankenstein et al., 2016, Gilboa and Schmeidler, 1989, Levy et al., 2010, Tymula et al., 2012). In this utility function, both the risk and the ambiguity of the options are considered, resulting in separate parameter estimates for risk tolerance and ambiguity aversion.

The expected utility, U is given by:

$$U\left(p,A,v\right)=\left(p-(\beta *p*A)\right)*v^{\rho }$$

In this model p is the probability of winning the high amount, v is the value of the high amount, A is the ambiguity level of the option. Risk tolerance is ρ. A risk neutral subject would have a ρ of 1 and would choose the option with the highest expected value. Risk seeking would be reflected in ρ> 1, resulting in a higher expected utility of the risky option compared to the expected value. Risk aversion would be reflected by ρ< 1. Ambiguity tolerance is β. An ambiguity neutral participant would assign the covered part of the bar proportionally to the chances of winning. That is, if the chance of winning is the high amount is 40%, an ambiguity neutral participant would assign 40% of the covered bar to the chance of winning the high amount, thereby essentially ignoring ambiguity, this would be reflected in a β of 1.^1^ An ambiguity averse participant would assign less than 40% of the covered bar to the chance of winning the high amount of money, which is reflected in positive values of β. Negative values of β would reflect ambiguity seeking.

The probability of choosing the risky choice option is then calculated using a softmax function:

$$P\left(\mathrm{chose}\;\mathrm{risky}\;\mathrm{lottery}\right)=\frac{{U_{\mathrm{risky}}}^{\left(1/\mu \right)}}{{U_{\mathrm{risky}}}^{\left(1/\mu \right)}+{U_{\mathrm{safe}}}^{\left(1/\mu \right)}}$$

In which μ is noise. As choices become more random, μ becomes larger. To test for any age-related differences in noise, we performed a model fitting procedure on the μ parameter fitted for each participant for each condition. We used non-linear mixed effects models to compare a total of three different models per condition. We compared a null model in which we did not include an age parameter, a model with a linear predictor for age testing for linear increases or decreases over age and a model with a quadratic predictor for age testing for adolescent-specific effects. Model fit was evaluated based on AIC values. Lower AIC values indicate a better fit. Model fit improvement was tested using a log likelihood ratio test. The best fitting model for the solo and social safe conditions was a null model indicating that there were no age-related differences in noise (AIC values solo condition null model: −52, linear age model: −50, quadratic age model: −48; AIC values social safe condition null model: −59, linear age model −57, quadratic age model −55). In the social risky condition, the best fitting model was a model including a quadratic regressor for age (AIC values social risky condition null model: 14, linear age model: 11, quadratic age model 9). In this model, there was a significant quadratic relationship with age in which μ was smallest in the mid-range of the age of the sample, i.e. 18 years old (B=0.53, t(61) = 2.16, p = .034), suggesting this age group showed the least randomness and thus most consistency in their choices.

Computational models were fitted using fmincon, search algorithm sqp, in MATLAB. Parameter initialization points were random with bounds 0–1. No parameters bounds for the solution were enforced. Models were fitted 50 times and the best model solution was selected based on log likelihood. Plots to illustrate the fit of the computational model on the raw data are shown in Supplementary Figure 3. Plots showing model fit for each individual participant are available on the Open Science Framework: https://osf.io/8frsj/. Parameter estimates were inspected for outliers. One participant selected the risky option on all lotteries in the social risky condition. Due to the lack of variation in the choice data, parameter estimates could not be established accurately and were therefore excluded from all analyses for this participant.

### 2.7 Risk and ambiguity tolerance comparisons between conditions and across age

We fitted computational models to derive risk and ambiguity attitudes in each of the three conditions (solo, social risky, social safe). Changes in these parameters would reflect changes in decision making strategies by social context and can therefore be used to parse out whether participants modify their choices in the social conditions due to changes in risk tolerance, ambiguity aversion, or both. To test whether risk and ambiguity attitudes were altered in the social conditions versus the solo condition, we fitted non-linear mixed effects models with a fixed effect for condition and a random effect for participant. Models were run using package nlme in R (Pinheiro et al., 2013; R Core Team, 2014). If a model with a fixed effect for condition fits significantly better than the null model without fixed effects, this would indicate that the variation in the dependent variable is significantly better explained when taking the condition into account.

We also fitted two additional mixed effects models to test for age related effects. We used the poly function in the nlme package (Pinheiro et al., 2013) in R to fit a model including a linear fixed effect of age and a model including both a linear and quadratic fixed effect of age. Linear effects of age would indicate a gradual increase or decrease with age, whereas a quadratic effect of age would indicate an age-range specific peak (or dip). Both of these models included a factor for condition (social safe vs. solo, or social risky vs. solo). We tested for interactions between the linear and quadratic fixed effects for age and condition. If models with an interaction for age fit significantly better than a model with only condition, this would indicate that the changes in the dependent variable between the conditions differed across age. Models were fit for the social risky condition vs solo and the social safe condition vs solo separately.

### 2.8 fMRI data analysis

FMRI preprocessing was performed using fmriprep, version 1.0.15 (Esteban et al., 2019). Preprocessing steps included slice-time correction, realignment, coregistration of functional to structural images and non-linear normalization of anatomical to MNI152 template space. Spatial smoothing using a 5 mm full width at half maximum Gaussian smoothing kernel was performed using fslmaths. FMRI analyses were performed using FSL, version 5.0.9 (Jenkinson et al., 2012). We used FEAT to estimate task effects (Woolrich et al., 2004). On the first level, regressors of interest were included for the solo condition, social risky condition, and social safe condition. Regressors were timelocked to the onset of the trials and duration was specified from the onset of the trial until the onset of the fixation cross. Not all trials shown in the solo condition were shown in both social information conditions. To permit creating contrasts that compare the same choice information in each condition (varying only in the social information), the trials in the solo condition were divided into three different categories: trials that were shown in the social risky condition, but not in the social safe condition; trials that were shown in the social safe condition, but not in the social risky condition; and trials that were shown in both conditions. A regressor of non-interest was included for missed trials. All regressors were convolved with a double-gamma hemodynamic response function included in FSL. Standard fMRIprep output nuisance regressors were included for 29 variables including motion and rotation in x, y, z directions, average csf signal and average white matter signal (see https://fmriprep.readthedocs.io/en/stable/outputs.html for full explanation of all nuisance regressors provided by fMRIPREP).

First level individual contrasts were then submitted to random effects group analyses using FLAME 1 + 2 as implemented in FSL. We used a threshold of z > 3.1, cluster corrected to FWE p < .05, for the group analyses. Parameter estimates were extracted from activated clusters to allow for descriptive plotting of the results. We first tested whether seeing choices of others was related to activation in neural regions previously found to be active in social contexts. We tested the whole brain contrast social>solo. As this contrast was intended to test for effects of social information regardless of the content of that information, we combined the social risky and social safe conditions. We then performed whole brain analyses to test which neural regions respond to the riskiness and ambiguity levels of the trials. These analyses were performed on the solo condition to isolate the effects of the riskiness and ambiguity of the trials, without the presence of social information.

To test which neural regions track the riskiness of the trials, we performed a parametric whole brain regression. We tested which neural regions responded to parametric increases in risk as well as which neural regions responded to parametric decreases in risk. Trials were categorized based on percentage chance of winning (20%; 40%; 60% and 80%). The *t*-test contrast assigned weights to these categories in a parametric manner. The positive linear contrast was coded as [−3; − 1; 1; 3] and the negative parametric test [3; 1; −1; −3]. To test which neural regions responded to the ambiguity level of the trials, we again used a parametric whole brain regression. Here we categorized trials based on the level of ambiguity (0%; 40%; 80%). The positive linear parametric contrast was coded as [−1; 0; 1] and the negative linear parametric contrast was coded as [1; 0; −1]. Exploratory analyses with age as a linear and quadratic regressor were performed and are available on NeuroVault: https://identifiers.org/neurovault.collection:10516.

To test how changes in risk and ambiguity attitudes are related to changes in neural activation, we used the results of the behavioral analyses to guide our analyses. In the behavioral analyses, we used computational models to test whether risk and ambiguity attitudes changed as a function of availability of social information. Differences in risk and ambiguity attitudes between the solo and social conditions were then used as regressors in a whole brain regression on the contrast of interest (i.e., social risky>solo or social safe>solo). Finally, we tested which neural regions showed age related differences in activation. We tested for linear increases and decreases in neural recruitment as well as quadratic effects in the contrast social>solo. We tested both a quadratic peak with age to identify neural regions that show highest levels of activation for the participants in the mid-range of age of the sample (i.e., 18 years of age) and a quadratic dip with age to identify neural regions that show lowest levels of activation for the mid-range of age in the sample. These are common patterns associating age and performance across many types of cognitive processes and will allow us to draw comparisons to a broad developmental literature when considering the trajectory of decision making in safe and risky contexts.

## 3 Results

### 3.1 Behavioral results

#### 3.1.1 Raw choice data

**Fig. 2:**
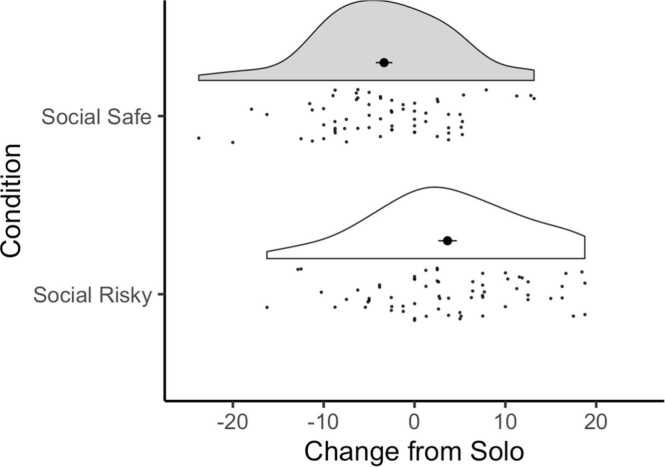
Raincloud plots displaying average change in percent choices for the risky option in the social safe and social risky condition compared to the matched trials in the solo condition. Black dots indicate the mean for each condition, error bars indicate standard error of the mean. Participants made significantly fewer risky choices in the social safe condition compared to the solo condition, and significantly more risky choices in the social risky condition compared to the solo condition.

To confirm that our social manipulation affected choice preferences, we tested whether participants changed the proportion of risky choices they selected in the social conditions compared to the solo condition. Participants selected the risky option, on average, on 42.49% of trials in the solo condition (SD_solo_=13.31, range_solo_=15–70), 70.41% of trials in the social risky condition (SD_social risky_=18.90, range_social risky_=32–100) and 15.74% of trials in the social safe condition (SD_social safe_=10.80, range_social safe_=1.67–46.67), see Fig. 2. In both social conditions, participants were presented with 40 unique lotteries (see Methods for details). To accurately reflect change in risky choices, we compared each social condition against a set of matching lotteries from the solo condition. One sample *t*-tests were run to test whether change in mean percentage risky choice was significantly different from zero. Mean percentage risky choice on matched trials in the solo condition was subtracted from mean percentage risky choice in each of the social conditions. Values above zero therefore indicate increases in risky choice, values below zero decreases in risky choice. In line with our hypothesis, participants chose the riskyoption significantly more often in the social risky condition than in the solo condition (t(64) = 3.56, p < .001). In the social safe condition, participants selected the risky option significantly less often than in the solo condition (t(64)=-3.69, p < .001).

#### 3.1.2 Computational models

**Fig. 3:**
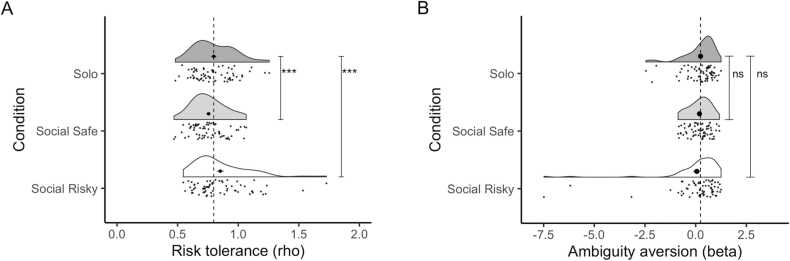
Raincloud plots displaying density and individual data points for the risk aversion parameter rho (panel A) and ambiguity aversion parameter beta (panel B) in the solo, social safe and social risky condition. Higher values for rho indicate higher tolerance to risk, higher values for beta indicate lower tolerance for ambiguity. Large black dot indicates the mean for each condition, error bars indicate standard error of the mean. Vertical dotted line indicates the mean in the solo condition, serving as a reference to indicate change compared to solo in the social conditions. The rho parameter (risk tolerance) is significantly lower for the social safe condition and significantly higher for the social risky condition, indicated by three asterisks (***), there are no significant differences in the beta parameter (ambiguity tolerance), indicated by ‘ns’.

We first fitted models to derive risk and ambiguity tolerance for each participant for the solo condition and each of the social information conditions. Changes in these parameters in the social conditions, compared to the solo condition, give insight to whether participants change their choices in the social information conditions due to changes in their risk or ambiguity attitudes, or both. After testing for differences between the conditions, we tested for age-related changes. For risk tolerance, the best fitting model for both conditions was a model with condition and no age effects, see Table 1 for AIC values for all models. This means that risk tolerance was significantly altered in both social conditions compared to the solo condition, and that the change in risk tolerance was not related to age. Participants showed increased risk tolerance in the social risky condition, (B=0.06, SE=0.021, t(63) = 2.55, *p* = .013), and a decrease in risk tolerance in the social safe condition (B=−0.04, SE=0.012, t(64) = −3.61, *p* < .001), see Fig. 3 A. For ambiguity aversion, the best fitting model was a null model, meaning there was no evidence for changes in ambiguity aversion in the social conditions compared to the solo condition (social risky condition vs solo condition: B=−0.19, SE=0.13, t(63) = −1.41, *p* = .164; social safe condition vs solo condition: B= −0.05, SE= 0.07, t(64) = −0.72, *p* = .476), see Fig. 3B. Also, we did not find age related differences in ambiguity aversion.

**Table 1:** Included fixed effects and AIC values for all models. Improvement of model fit was tested using a log likelihood ratio test. Models were regarded statistically better if they exceeded the threshold of p < .05. Preferred models are depicted in bold. A full description of the statistical parameters for each of these models is available on the Open Science Framework: https://osf.io/8frsj/.

Fixed effects	Social risky vs solo		Social safe vs solo	
	Risk tolerance (rho)	Ambiguity aversion (beta)	Risk tolerance (rho)	Ambiguity aversion (beta)
None (null model)	-70	**383**	-170	**234**
Condition	**-75**	383	**-180**	236
Condition * linear age	-72	387	-176	238
Condition * linear age & Condition * quadratic age	-71	388	-176	240

### 3.2 FMRI results

#### 3.2.1 Main effects

**Fig. 4:**
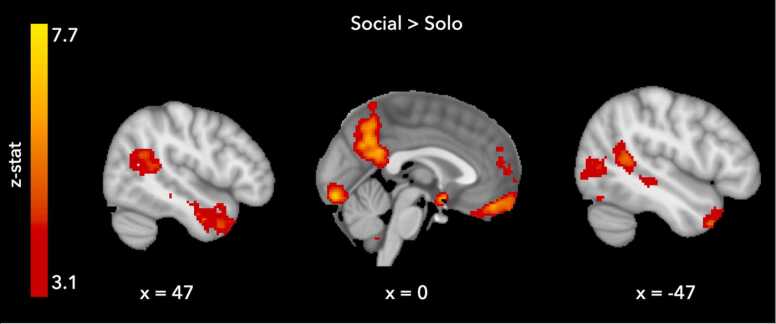
Whole brain results for both social conditions combined vs solo. Whole brain statistical maps were thresholded at z > 3.1, p < .05 FWE corrected, MNI coordinates are provided for each slice.

We first tested which neural regions were active when participants were presented with information about others’ choices. As expected, results for this social>solo whole brain contrast showed activation in a set of regions previously related to social information processing including the ventromedial prefrontal cortex, temporoparietal junction (TPJ) and precuneus, see Fig. 4 and Table 2. We then performed separate analyses to test which neural regions showed increases in activity in response to increases in risk and ambiguity. Whole brain results for the parametric regression testing for increased activation with increasing riskiness of the trials resulted in activation in regions including the ventral striatum and anterior cingulate cortex, see Table 3. A parametric regression for decreasing risk resulted in activation in the occipital cortex, see Table 3. Whole brain parametric regression results for the ambiguity analysis showed increased activation in a distributed set of regions across the brain including the precuneus and superior frontal gyrus for parametric increases in ambiguity, and increased activation in the temporal pole and supramarginal gyrus for parametric decreases in ambiguity, see Table 4 for all clusters of activation for both analyses.

**Table 2:** Whole brain table for activation for the contrast social (i.e. social risky & social safe) > solo. Peak coordinates for each cluster are reported as well as lateralization of the effect (L=left; R=right). Whole brain results are thresholded at z > 3.1, p < .05 FWE corrected.

Region	z-stat	L/R		MNI		voxels
			x	y	z	
Occipital Cortex	7.70	L	-6	-82	-18	3492
Ventromedial Prefrontal Cortex	6.02	R	0	48	-20	1799
Precuneus	6.97	R	6	-52	20	2136
Temporal Parietal Junction	5.36	L	-58	-46	2	2110
Temporal Parietal Junction	5.76	R	44	-48	18	869
Temporal Pole	6.43	R	58	-2	-28	1584
Temporal Pole	5.28	L	-44	16	-38	733
Ventral Striatum	6.04	R	0	4	-14	117
Cerebellum	5.73	L	-6	-56	-46	235
Cerebellum	4.65	R	20	-76	-36	104
Cerebellum	6.01	L	-26	-78	-36	224

**Table 3:** Neural regions showing increased activation for the parametric contrast for increasing risk and decreasing risk. Peak coordinates for each cluster are reported as well as lateralization of the effect (L=left; R=right). Whole brain activation maps were thresholded at z > 3.1, p < .05 FWE corrected.

Region	z-stat	L/R		MNI		voxels
			x	y	z	
*Parametric whole brain contrast for increasing risk*						
Supramarginal Gyrus	4.28	L	-54	-46	50	143
Ventral Striatum	4.41	R	14	10	-6	127
Ventral Striatum	4.42	L	-10	10	-6	117
Precuneus	4.34	R	26	-52	22	90
Precuneus	4.55	R	8	-54	6	72
Anterior Cingulate Cortex	3.92	R	6	36	4	68
Fusiform Gyrus	4.09	R	26	-70	-4	67
*Parametric whole brain contrast for decreasing risk*						
Occipital Cortex	4.77	L	-32	-92	-2	995
Occipital Cortex	4.90	R	40	-86	-12	446
Occipital Cortex	3.88	R	38	-86	12	76

**Table 4:** Neural regions showing increased activation for the parametric contrasts for increasing ambiguity and decreasing ambiguity levels of the trials. Peak coordinates for each cluster are reported as well as lateralization of the effect (L=left; R=right). Whole brain activation maps were thresholded at z > 3.1, p < .05 FWE corrected.

Region	z-stat	L/R		MNI		voxels
			x	y	z	
*Parametric whole brain contrast for increasing ambiguity*						
Occipital Cortex	8.54	L	-20	-98	-2	7482
Superior Temporal Cortex	4.33	R	60	-18	28	221
Superior Frontal Gyrus	4.19	R	26	4	54	121
Precuneus	3.82	R	4	-66	52	77
Inferior Parietal Cortex	4.13	L	-58	-26	32	76
*Parametric whole brain contrast for decreasing ambiguity*						
Occipital Cortex	4.29	L	-32	-78	-40	258
Temporal Pole	4.58	L	-44	16	-14	245
Posterior Cingulate Gyrus	4.44	R	2	-36	32	173
Supramarginal Gyrus	3.97	R	60	-42	0	129
Superior Temporal cortex	4.12	R	52	-10	-22	83
Ventromedial Prefrontal cortex	4	R	0	50	-6	78
Ventromedial Prefrontal cortex	3.62	L	-10	46	38	73
Supramarginal Gyrus	4.22	L	-64	-26	-24	71

#### 3.2.2 Whole brain regressions

**Fig. 5:**
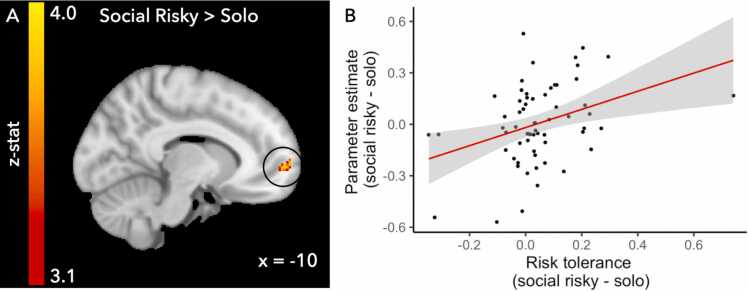
Panel A) Whole brain regression analysis on the contrast Social Risky-Solo with the change in risk tolerance between the Social Risky and Solo condition (rho_social risky_ – rho_solo_). Panel B) Scatter plot representing the relationship between the change in risk tolerance and activation in the left medial prefrontal cortex cluster (MNI coordinates for the peak voxel: x = −10 y = 60 z = 0) circled in panel A. Note that this plot is included for visualization purposes only. Repeating the analysis with exclusion of one extreme datapoint resulted in activation in the same neural region.

The next set of analyses were guided by the behavioral results showing a change in risk tolerance, but not ambiguity tolerance, in the social conditions compared to the solo condition. We tested how changes in risk tolerance were related to changes in neural activation by performing a whole brain regression on the social risky>solo and social safe>solo contrasts using the difference in risk tolerance in the respective social condition and solo condition (rho_social_ – rho_solo_) as a regressor. We performed separate whole brain regressions for the contrast social risky>solo and social safe>solo to ensure matching of the risk and ambiguity levels for the social and solo conditions. We tested for both positive linear and negative linear effects. The positive linear whole brain regression for the contrast social risky>solo showed a cluster in the left medial prefrontal cortex (see Fig. 5 and Table 5). Thus, neural activation in this cluster was higher for those participants who showed a higher difference in risk tolerance between the social risky and solo condition. No clusters reached significance for other whole brain regressions.

**Table 5:** Neural regions showing activation for the whole brain regressions. Peak coordinates for each cluster are reported as well as lateralization of the effect (L=left; R=right). Whole brain results are thresholded at z > 3.1, p < .05 FWE corrected.

Regression	Region	z-stat	L/R		MNI		voxels
				x	y	z	
*Social risky > Solo whole brain regressions with rho*_*social*_*- rho*_*solo*_							
Positive linear	Medial Prefrontal Cortex	4.10	L	-10	60	0	80
*Social > Solo whole brain regressions with age*							
Positive quadratic (ie. peak)	Temporal Parietal Junction	3.98	R	54	-40	18	97
Negative quadratic (i.e. dip)	Ventrolateral Prefrontal Cortex	3.89	L	-42	42	2	126

#### 3.2.3 Age related differences in neural activation

Lastly, we tested for age-related differences in neural activation. Computational analyses did not show age related associations with estimated parameters. However, it is possible that participants of different ages would show different neural recruitment to reach the same behavioral outcome. Therefore, we performed exploratory whole brain regressions on the contrast social>solo. We tested for linear increases and decreases with age, as well as quadratic effects showing elevated or decreased neural activation. Given the age range in the current study and previous work showing adolescent-specific patterns of responses in the age range 16–18 years for reward sensitivity (Braams et al., 2015, Schreuders et al., 2018), social evaluation (Somerville et al., 2013) and social information (Braams et al., 2019), we tested quadratic patterns of responses with a peak/dip around the average age of the sample (i.e., approximately 18 years old).

**Fig. 6:**
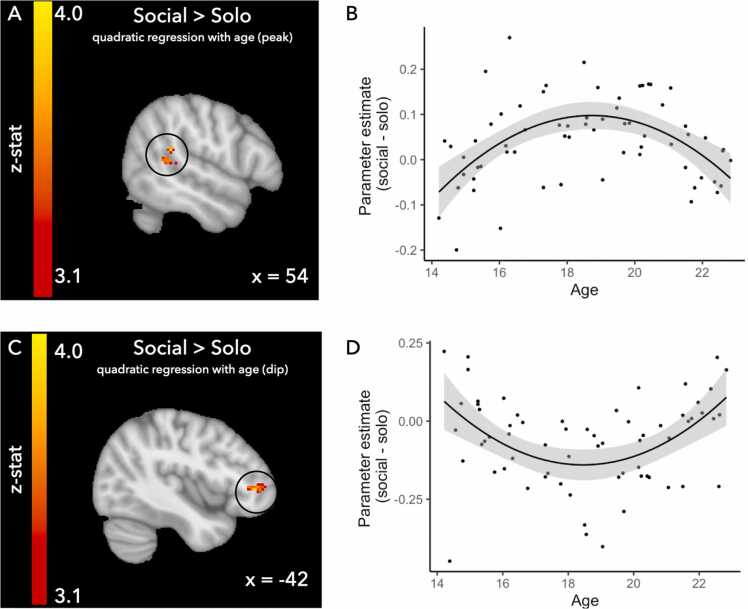
Quadratic whole brain regressions on the social (social risky & social safe) - solo contrast and visualization of the results. Whole brain regressions were thresholded at z > 3.1, p < .05 FWE corrected, see also Table 5. Panel A & B: positive quadratic regression resulting in activation in the right temporal parietal junction (TPJ). Panel C & D: negative quadratic regression resulting in activation in the left ventrolateral prefrontal cortex, note that these plots are included for visualization purposes only.

Results showed that the mid-range of the ages in the sample showed the greatest activation magnitude in the temporoparietal junction (see Fig. 6 and Table 5) and that this age range showed the lowest magnitude of neural activation in the ventrolateral prefrontal cortex (see Fig. 6 and Table 5). Results did not show any linear effects, positive or negative, with age.

## 4Discussion

The current study tested how information about others’ choices induced changes in decision making under uncertainty. We utilized a computational modeling framework to assess how participants’ risk and ambiguity attitudes change as a function of seeing information about others’ choices. Functional MRI data were collected to test neural activation related to behavioral effects. To test for age related differences, we included participants between the ages of 14–22 years old.

Behavioral results showed that participants followed choices of others in both risky and safe directions. We did not find evidence for age-related modulation of the tendency to align with choices of others for either of the social conditions. Computational modeling analyses showed that aligning one’s own choices with others was related to changes in risk attitude, but not ambiguity attitude. On the neural level, the change in risk tolerance was positively correlated with activation in the left medial prefrontal cortex. Exploratory analyses of age-related patterns resulted in adolescent specific patterns of responses in the right TPJ and left ventrolateral prefrontal cortex. Together, these results suggest that changes in risky choice behavior as a function of information about others’ previous choices are induced by changes in the risk perception and valuation of the different choice options. These results show that information about others’ choices changes individuals’ choice behavior. By showing how information about others’ choices independently influences risk and ambiguity attitudes its neural correlates, we contribute to a more detailed account of the nature of social influence based on the choice history of peers and reveal consistent use of this information across age from adolescence to young adulthood. Together, these results provide more insight into the complex effects of social information on risky choices.

### 4.1 Behavioral findings

We first tested whether information about others’ previous choices induced changes in participants’ risky choices. As expected and in line with previous work (Braams et al., 2019, Chung et al., 2015), participants made more risky choices when they saw that a previous participant made a risky choice, and fewer risky choices when the previous participant made a safe choice. Contrary to a previous study using a very similar task design, we did not find evidence for age related effects (Braams et al., 2019). One difference between these two studies is that the current study used a slightly narrower age range in which we tested participants aged 14–22, whereas the previous study tested participants aged 12–22.

Results showed that when participants saw a previous participant select the risky option, they showed more tolerance for risk, but not ambiguity. A similar pattern was observed for the condition in which participants saw a safe choice of a previous participants. Here participants showed less tolerance for risk, but no changes in the ambiguity parameter. These results are in line with previous work using a similar paradigm (Blankenstein et al., 2016). The observed changes in the risk parameter imply that information about others’ previous choices attributes a higher subjective value to that option, which increases the difference in subjective value for the two choice options and ultimately leading participants to select that option more often.

It is possible that ambiguity attitudes are less susceptible to peer influence than risk attitudes. There are several possible explanations why participants’ ambiguity tolerance was not affected by social information. One possibility is that ambiguity attitudes do not change because participants do not regard the peer as more knowledgeable than they are. Indeed, in the task context, participants are instructed that peers had the same information they did – not unique information that could help resolve the ambiguity of the participant’s decision. Although previous research has shown that expert opinions exert a strong influence on choice behavior (Meshi et al., 2012) and that adolescents are influenced by the opinion of an expert to a larger degree than adults (Engelmann et al., 2012), the present experimental context could have attenuated this effect as peers did not have unique expertise regarding the choice at hand. An alternative experimental context could alter the instructions so that participants believe the peer has knowledge they did not, which we predict would induce participants to follow peers’ choices in the ambiguous conditions. Another possibility is that the change in ambiguity aversion could be so small that it was not detected at the current power level. Future work could extend the current study and use a larger sample to test for more subtle changes in ambiguity aversion than were tested in the current study.

### 4.2 fMRI findings

On the neural level we first confirmed that the social condition elicited activation in neural regions previously related to social information processing. We found higher activation in a collection of brain regions including the precuneus, bilateral TPJ, bilateral temporal pole, and medial prefrontal cortex in the social conditions versus the solo condition. These areas are well known for their engagement in social processes (Blakemore, 2008, Van Overwalle, 2009). These findings are in line with expectations that participants process social information when they are presented with information about others’ choices. We then tested which neural regions responded to increased risk and ambiguity of the choice options. As expected, results showed increased activation in a set of neural regions including the ventral striatum and anterior cingulate cortex when the riskiness of the choice options increased (Levy, 2017, Poudel et al., 2020). Increasing ambiguity of the choice options was related to increased activation in a set of regions including the precuneus and superior frontal gyrus. These results correspond with two meta-analyses comparing risky and ambiguous decision making (Krain et al., 2006, Poudel et al., 2020).

After establishing that our experiment elicited the expected neural responses for the social manipulation and the risk and ambiguity levels of the choice options, we used the results on the behavioral level and tested which neural regions showed responses related to changes in risk tolerance. We showed that participants who showed higher changes in risk tolerance when seeing a risky choice of someone else also show increased activation in the left medial prefrontal cortex. The medial prefrontal cortex is an area known for both integration of information from multiple sources as well as processing of social information (Burke et al., 2013, Somerville et al., 2013, Somerville et al., 2010, van den Bos et al., 2007, Van Overwalle, 2009). Although interpretations based on neural activity should be made with caution, it appears as if participants employ the medial prefrontal cortex to integrate information about their own risk preferences and the information they receive about others’ risk preferences to make their final choice. In other words, the subjective value of the risky option is constructed from both social information and participants’ private risk preferences.

In the current study, we did not find age related differences in magnitude of change in risk tolerance between the social and solo conditions. Also, we did not find any age-related differences in the medial prefrontal cortex activation. Adolescents show increased risk taking behavior in the presence of their peers in the real world (Brechwald and Prinstein, 2011, Simons-Morton et al., 2005). This is mirrored by previous studies in the lab showing that adolescents make more risky choices when their peers are present (Albert et al., 2013, Chein et al., 2011, Gardner and Steinberg, 2005). Together, these observations led to the conclusion that adolescents are prone to more risk taking behavior when peers are involved.

However, here we do not find evidence for differences in social influence on choice behavior between adolescents and adults, neither for behavioral performance nor in neural activity. Although the absence of statistical significance must be interpreted with caution, these results could support the interpretation that adolescents are influenced by the choices of peers to a similar extent as adults are. In other words, information about previous choices from similar aged peers holds as much sway for young adults as adolescents.

Social influence is an important and complex construct, operationalized in many different ways experimentally. One study directly testing the difference between peer observation and mere peer presence without observation showed that only in the active peer observation condition adolescents changed their behavior compared to an alone condition (Somerville et al., 2019). In the current study, we used operationalized social influence as information about previous choices of others and the choices made by the participant were not observed by a peer. Based on the results of the current study and the study by Somerville et al. (2019) we speculate that active observation of peers, which was not used in the present study, may be a key factor for increases in risky decision making. Possibly, the effect of direct observation on risky choices might be related to the (assumed) positive reputational effects of risk taking, which remains an important topic for future work.

Exploratory age-related analyses to test for linear and quadratic patterns of neural responses showed quadratic patterns in the TPJ and ventrolateral prefrontal cortex. We found a quadratic pattern of neural responses with the highest neural activation in the right TPJ for the 18 year olds in the sample, and the lowest neural activation in the left ventrolateral prefrontal cortex for this age group. The TPJ is a region which is related to processing of social information (Blakemore, 2008, Van Overwalle, 2009). The lateral parts of the prefrontal cortex are related to inhibitory processing (Aron et al., 2004, Munakata et al., 2011, Wagner et al., 2001). However, activation in these regions was not related to a behavioral correlate. Future work should test how unique patterns of neural responses in these regions are related to behavior.

### 4.3 Limitations

The current study tested how information about others’ choices influences risky decision making. Although information about others’ choices is a form of peer influence that occurs in the real world, the current peer manipulation shows different effects compared to other forms of peer influence such as peer monitoring or active engagement of the peer. Most adolescent risk-taking behavior in the real world occurs when individuals are observed or their behavior is encouraged by their peers. It is likely that these types of peer influence induce behavioral changes with different mechanisms than the current peer manipulation. Future work should test whether other forms of peer influence have similar effects on behavior and its neural correlates. Secondly, in the current study we used unknown peers. Although the use of unknown peers ensures that the manipulation is consistent for all participants, this is at the expense of ecological validity. Future work should test how information about choices of real peers influences behavior. Thirdly, in the current study we tested for linear and quadratic patterns of age-related change. Although these patterns are commonly used to detect age-related changes, linear and quadratic models limit the detection power of more complex age-related patterns. Future work could use more complex and/or data driven methods such as generalized additive modeling to identify nonlinear age-related changes. Lastly, in the current study we tested a cross-sectional sample allowing for between subject tests of age-related change. To test how decision making changes over age within the individual, future work should collect longitudinal data. Moreover, a longitudinal approach might benefit from sampling an age range which begins younger and extends older than the sample tested here. Here, we focused on ages in which we expected the greatest effects from information about others’ previous choices. Extending this understanding to include periods in which others’ choices are less influential – possibly younger and/or older ages – would be useful to informing windows of differential influence of social factors in decision making. Finally, a larger sample size could be more sensitive to detecting smaller effect sizes in behavioral and neural responses.

## 5 Conclusion

The current study aimed to evaluate how information about others’ choices affects risk and ambiguity attitudes in adolescents and adults. We found that information about others’ choices affects risk tolerance, but did not find evidence that others’ choices affects ambiguity aversion. On the neural level, the changes in risk tolerance were positively related to changes in medial prefrontal cortex activation, a region believed to encode the subjective value of choice options. We did not find evidence for age-related differences in these processes. Although the findings should be interpreted with caution, they suggest that adolescents use previous choice information similarly to adults. Together with prior work demonstrating elevated social influence in adolescence, these findings highlight the complexity of the construct of peer influence.

## CRedit authorship contribution statement

**Barbara R. Braams:** Conceptualization, Funding acquisition, Investigation, Methodology, Formal analysis, Writing – original draft Preparation, Writing – review & editing, Visualization. **Juliet Y. Davidow:** Methodology, Writing – review & editing. **Leah H. Somerville:** Conceptualization, Funding acquisition, Supervision, Writing – review & editing.

## Declaration of Competing Interest

The authors declare that they have no known competing financial interests or personal relationships that could have appeared to influence the work reported in this paper.

## Data Availability

Unthresholded statistical maps for whole brain contrasts are available on NeuroVault (https://identifiers.org/neurovault.collection:10516). This collection includes whole brain contrasts for social vs solo, and the parametric analysis for risk and ambiguity, and linear and quadratic regressions with age on these contrasts. The task and analysis scripts are available on https://osf.io/8frsj/. Due to the nature of the data and restrictions imposed by the Committee on the Use of Human Subjects at Harvard University we are not able to publicly share the data. However, data will be made available to researchers upon reasonable request.

## Footnotes

1 Note that this is a generalized version of the formula previously used in Tymula et al. (2012) in which all ambiguous lotteries had an underlying probability of winning of 50%, resulting in p * A.5 *A and simplified to $\frac{A}{2}$.

## References

[bib1] Albert D., Chein J., Steinberg L. (2013). Peer influences on adolescent decision making. Curr. Dir. Psychol. Sci..

[bib2] Aron A.R., Robbins T.W., Poldrack R.A. (2004). Inhibition and the right inferior frontal cortex. Trends Cogn. Sci..

[bib3] Bartra O., McGuire J.T., Kable J.W. (2013). The valuation system: a coordinate-based meta-analysis of BOLD fMRI experiments examining neural correlates of subjective value. Neuroimage.

[bib4] Blakemore S.J. (2008). The social brain in adolescence. Nat. Rev. Neurosci..

[bib5] Blankenstein N.E., Crone E.A., van den Bos W., van Duijvenvoorde A.C.K. (2016). Dealing with uncertainty: testing risk- and ambiguity-attitude across adolescence. Dev. Neuropsychol..

[bib6] Blankenstein N.E., Peper J.S., Crone E.A., van Duijvenvoorde A.C.K. (2017). Neural mechanisms underlying risk and ambiguity attitudes. J. Cogn. Neurosci..

[bib7] Blankenstein N.E., Schreuders E., Peper J.S., Crone E.A., van Duijvenvoorde A.C.K. (2018). Individual differences in risk-taking tendencies modulate the neural processing of risky and ambiguous decision-making in adolescence. Neuroimage.

[bib8] Braams B.R., Davidow J.Y., Somerville L.H. (2019). Developmental patterns of change in the influence of safe and risky peer choices on risky decision-making. Dev. Sci..

[bib9] Braams B.R., van Duijvenvoorde A.C.K., Peper J.S., Crone E.A. (2015). Longitudinal changes in adolescent risk-taking: a comprehensive study of neural responses to rewards, pubertal development, and risk-taking behavior. J. Neurosci..

[bib10] Brechwald W.A., Prinstein M.J. (2011). Beyond homophily: a decade of advances in understanding peer influence processes. J Res. Adolesc..

[bib11] Burke C.J., Brunger C., Kahnt T., Park S.Q., Tobler P.N. (2013). Neural integration of risk and effort costs by the frontal pole: only upon request. J. Neurosci..

[bib12] Chein J., Albert D., O’Brien L., Uckert K., Steinberg L. (2011). Peers increase adolescent risk taking by enhancing activity in the brain’s reward circuitry. Dev. Sci..

[bib13] Chung D., Christopoulos G.I., King-Casas B., Ball S.B., Chiu P.H. (2015). Social signals of safety and risk confer utility and have asymmetric effects on observers’ choices. Nat. Neurosci..

[bib14] Chung D., Orloff M.A., Lauharatanahirun N., Chiu P.H., King-Casas B. (2020). Valuation of peers’ safe choices is associated with substance-naïveté in adolescents. Proc. Natl. Acad. Sci..

[bib15] Cialdini R.B., Goldstein N.J. (2004). Social influence: compliance and conformity. Ann. Rev. Psychol..

[bib16] Ciranka S., van den Bos W. (2021). Adolescent risk-taking in the context of exploration and social influence. Dev. Rev..

[bib17] Clithero J.A., Rangel A. (2014). Informatic parcellation of the network involved in the computation of subjective value. Soc. Cogn. Affect. Neurosci..

[bib18] Davidow J.Y., Sheridan M.A., Van Dijk K.R.A., Santillana R.M., Snyder J., Vidal Bustamante C.M., Somerville L.H. (2019). Development of prefrontal cortical connectivity and the enduring effect of learned value on cognitive control. J. Cogn. Neurosci..

[bib19] Decker J.H., Lourenco F.S., Doll B.B., Hartley C.A. (2015). Experiential reward learning outweighs instruction prior to adulthood. Cogn. Affect. Behav. Neurosci..

[bib20] Doherty S.T., Andrey J.C., MacGregor C. (1998). The situational risks of young drivers: the influence of passengers, time of day and day of week on accident rates. Accid. Anal. Prev..

[bib21] Eaton D.K., Kann L., Kinchen S., Shanklin S., Ross J., Hawkins J. (2010). Youth risk behavior surveillance - United States, 2009. MMWR Surveill. Summ..

[bib22] Ellsberg D. (1961). Risk, ambiguity, and the savage axioms. Q. J. Econ..

[bib23] Engelmann J.B., Moore S., Capra C.M., Berns G.S. (2012). Differential neurobiological effects of expert advice on risky choice in adolescents and adults. Soc. Cogn. Affect. Neurosci..

[bib24] Esteban O., Markiewicz C.J., Blair R.W., Moodie C.A., Isik A.I., Erramuzpe A., Snyder M. (2019). fMRIPrep: a robust preprocessing pipeline for functional MRI. Nat. Methods.

[bib25] Gardner M., Steinberg L. (2005). Peer influence on risk taking, risk preference, and risky decision making in adolescence and adulthood: an experimental study. Dev. Psychol..

[bib26] Gilboa I., Schmeidler D. (1989). Maxmin expected utility with non-unique prior. J. Math. Econ..

[bib27] Green P., MacLeod C.J. (2016). SIMR: an R package for power analysis of generalized linear mixed models by simulation. Method Ecol. Evol..

[bib28] Huettel S.A., Stowe C.J., Gordon E.M., Warner B.T., Platt M.L. (2006). Neural signatures of economic preferences for risk and ambiguity. Neuron.

[bib29] Jenkinson M., Beckmann C.F., Behrens T.E., Woolrich M.W., Smith S.M. (2012). Fsl. Neuroimage.

[bib30] Kable J.W., Glimcher P.W. (2007). The neural correlates of subjective value during intertemporal choice. Nat. Neurosci..

[bib31] Krain A.L., Hefton S., Pine D.S., Ernst M., Xavier Castellanos F., Klein R.G., Milham M.P. (2006). An fMRI examination of developmental differences in the neural correlates of uncertainty and decision‐making. J. Child Psychol. Psychiatry.

[bib32] Levy I. (2017). Neuroanatomical substrates for risk behavior. Neuroscientist.

[bib33] Levy I., Snell J., Nelson A.J., Rustichini A., Glimcher P.W. (2010). Neural representation of subjective value under risk and ambiguity. J. Neurophysiol..

[bib34] Meshi D., Biele G., Korn C.W., Heekeren H.R. (2012). How expert advice influences decision making. PLoS One.

[bib35] Munakata Y., Herd S.A., Chatham C.H., Depue B.E., Banich M.T., O’Reilly R.C. (2011). A unified framework for inhibitory control. Trends Cogn. Sci..

[bib36] Pinheiro, J., Bates, D., DebRoy, S., Sarkar, D., & Team, R.C. (2013). nlme: Linear and nonlinear mixed effects models. R package version, 3(1), 111.

[bib37] Poudel R., Riedel M.C., Salo T., Flannery J.S., Hill-Bowen L.D., Eickhoff S.B., Sutherland M.T. (2020). Common and distinct brain activity associated with risky and ambiguous decision-making. Drug Alcohol Depend..

[bib38] Core Team R. (2014). Austria: R Foundation for Statistical Computing.

[bib39] Schreuders E., Braams B.R., Blankenstein N.E., Peper J.S., Güroğlu B., Crone E.A. (2018). Contributions of reward sensitivity to ventral striatum activity across adolescence and early adulthood. Child Dev..

[bib40] Siegel J.S., Power J.D., Dubis J.W., Vogel A.C., Church J.A., Schlaggar B.L., Petersen S.E. (2014). Statistical improvements in functional magnetic resonance imaging analyses produced by censoring high-motion data points. Hum. Brain Mapp..

[bib41] Simons-Morton B., Lerner N., Singer J. (2005). The observed effects of teenage passengers on the risky driving behavior of teenage drivers. Accid Anal. Prev..

[bib42] Somerville L.H., Haddara N., Sasse S.F., Skwara A.C., Moran J.M., Figner B. (2019). Dissecting “peer presence” and “decisions” to deepen understanding of peer influence on adolescent risky choice. Child Dev..

[bib43] Somerville L.H., Jones R.M., Ruberry E.J., Dyke J.P., Glover G., Casey B.J. (2013). The medial prefrontal cortex and the emergence of self-conscious emotion in adolescence. Psychol. Sci..

[bib44] Somerville L.H., Kelley W.M., Heatherton T.F. (2010). Self-esteem modulates medial prefrontal cortical responses to evaluative social feedback. Cereb. Cortex..

[bib45] Tversky A., Kahneman D. (1979). Prospect theory: an analysis of decision under risk. Econometrica.

[bib46] Tversky A., Kahneman D. (1992). Advances in prospect-theory - cumulative representation of uncertainty. J. Risk Uncertain..

[bib47] Tymula A., Rosenberg Belmaker L.A., Roy A.K., Ruderman L., Manson K., Glimcher P.W., Levy I. (2012). Adolescents’ risk-taking behavior is driven by tolerance to ambiguity. Proc. Natl. Acad. Sci. U.S.A..

[bib48] van den Bos W., Bruckner R., Nassar M.R., Mata R., Eppinger B. (2018). Computational neuroscience across the lifespan: Promises and pitfalls. Dev. Cogn. Neurosci..

[bib49] van den Bos W., McClure S.M., Harris L.T., Fiske S.T., Cohen J.D. (2007). Dissociating affective evaluation and social cognitive processes in the ventral medial prefrontal cortex. Cogn. Affect. Behav. Neurosci..

[bib50] van Hoorn J., Fuligni A.J., Crone E.A., Galvan A. (2016). Peer influence effects on risk-taking and prosocial decision-making in adolescence: insights from neuroimaging studies. Curr. Opin. Behav. Sci..

[bib51] Van Overwalle F. (2009). Social cognition and the brain: a meta-analysis. Hum. Brain Mapp..

[bib52] Von Gaudecker H., Van Soest A., Wengstrom E. (2011). Heterogeneity in risky choice behavior in a broad population. Am. Econ. Rev..

[bib53] Wagner A.D., Maril A., Bjork R.A., Schacter D.L. (2001). Prefrontal contributions to executive control: fMRI evidence for functional distinctions within lateral prefrontal cortex. Neuroimage.

[bib54] Wilson, R., Collins, A. , 2019. Ten simple rules for the computational modeling of behavioral data. arxiv. In. 10.7554/eLife.49547 PMC6879303 31769410

[bib55] Woolrich M.W., Behrens T.E.J., Beckmann C.F., Jenkinson M., Smith S.M. (2004). Multilevel linear modelling for FMRI group analysis using Bayesian inference. Neuroimage.

[bib56] Zaki J., Kallman S., Wimmer G.E., Ochsner K., Shohamy D. (2016). Social cognition as reinforcement learning: feedback modulates emotion inference. J.Cogn. Neurosci..

[bib57] Zaki J., Schirmer J., Mitchell J.P. (2011). Social influence modulates the neural computation of value. Psychol. Sci..

