# Supplementary material for: Information about others’ choices selectively alters risk tolerance and medial prefrontal cortex activation across adolescence and young adulthood
Source: Dev Cogn Neurosci. 2021 Nov 18;52:101039. doi: 10.1016/j.dcn.2021.101039 (PMC8607164; doi:10.1016/j.dcn.2021.101039)
Supplement: Supplementary file 1 — Supplementary material [file mmc1.docx]

# Supplementary materials

## Choice simulations

To assure that the choices of the other participants were believable, we simulated choices using a computational model, see Behavioral Data Analysis section for a description of the model. We aimed to simulate choices for one peer with a more risk tolerant attitude (risky peer) and one peer with a less tolerant risk attitude (safe peer). Choices were simulated such that the risky peer selected the risky option on 75% of the trials and the safe peer selected the risky option on 25% of the trials. To simulate the choices, we used different risk aversion and ambiguity aversion parameters for the risky and the safe peer. Risk aversion is reflected as $\rho$. A $\rho$ of 1 would indicate risk neutrality, $\rho$ >1 would indicate risk seeking and $\rho$ <1 risk aversion. Ambiguity aversion is reflected as $\beta$. A $\beta$ of zero would indicate ambiguity neutrality, positive values of $\beta$ indicate ambiguity aversion and negative values indicate ambiguity tolerance. Choices for the risky peer were simulated with a $\rho$ of .9 and $\beta$ of -.3 and choices for the safe peer were simulated with a $\rho$ of .7 and $\beta$ of .1, the noise parameter was set to .5 for both peers. The odds of choosing the risky option increased when the difference between the expected value of the safe option (always $5) and the risky option (variable between $1 and $60) increases. As risk aversion increases, the differences in expected value needs to be larger for an individual to select the risky option. The difference in expected value between the safe and risky option is calculated by subtracting the expected value of the safe option from the expected value of the risky option. Positive values indicate a higher expected value for the risky option, negative values indicate a higher expected value of the safe option. The average difference between the expected value of the safe and risky option on which the risky peer selected the risky option was $15.60 (range $0-$55) and $26.62 (range $6.80-$55) for the safe peer. The average difference between the expected value of the safe and risky option on which the risky peer selected the safe option was -$2.00 (range -$4.00 to $1.40) and $1.28 (range -$4.00 to $10.20) for the safe peer.

If the expected value of the risky choice option was lower than $5 (i.e., the expected value of the safe option), simulated choices always resulted in a safe choice. Consequently, these choice options were not presented in the social risky condition. When the expected value for the risky choice option was higher than $16.20, simulated choices always resulted in a risky option. Consequently, these lotteries were not presented in the social safe condition. The number of unique lotteries in each social condition was 40, some of these lotteries were presented twice since participants made choices on 60 lotteries in both social conditions. Note that although the ‘peers’ both showed a distinct decision making profile (i.e., risky or safe) participants saw 50% risky choices of the peers and 50% safe choices in aggregate.

**Peer ratings**

Participants rated supposed other participants on seven dimensions before they completed the task. Participants then selected two individuals for whom they wanted to see their choices in the task. Average ratings on all dimension for the selected peers and non-selected peers are shown in Supplementary Figure 1. Selected peers were rated significantly higher, than non-selected peers for all dimensions (all t’s(63)>3.52, p’s<.001, corrected for multiple comparisons). Ratings for one participant were not included due to a technical error.

After participants selected the two peers, these peers were randomly assigned to be the risky and safe peer. As expected, the ratings, collected before the onset of the task, between these two peers was not statistically different for any of the measured dimensions (see Supplementary Figure 2). Ratings of the probability of becoming friends with safe peer were rated slightly lower than ratings of the probability to become friends with the risky peer. However, this difference did not reach significance after correcting for multiple comparisons (t(63)=2.23, p=.029), see Supplementary Figure 2. Furthermore, simple linear regressions indicated that the relationship between the rating of probability of becoming friends was not related to how much participants were influenced by the choices of the safe peer (F(1,62)=2.21, p=.14) or the risky peer (F(1,62)=.038, p=.846). There was no relationship between any of the other ratings and behavior of the participants (all F’s(1,62)<3.31, all p’s>.074).

**
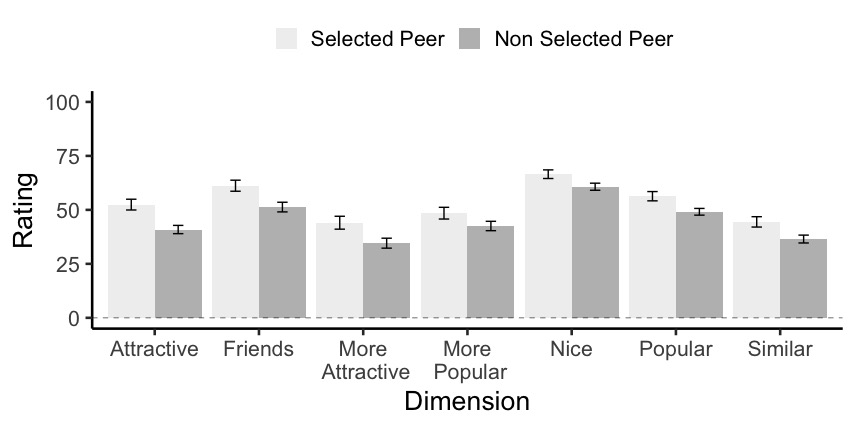
**

*Supplementary Figure 1.* Average ratings on all dimension for the selected peers and non-selected peers. Ratings were selected before the onset of the task.


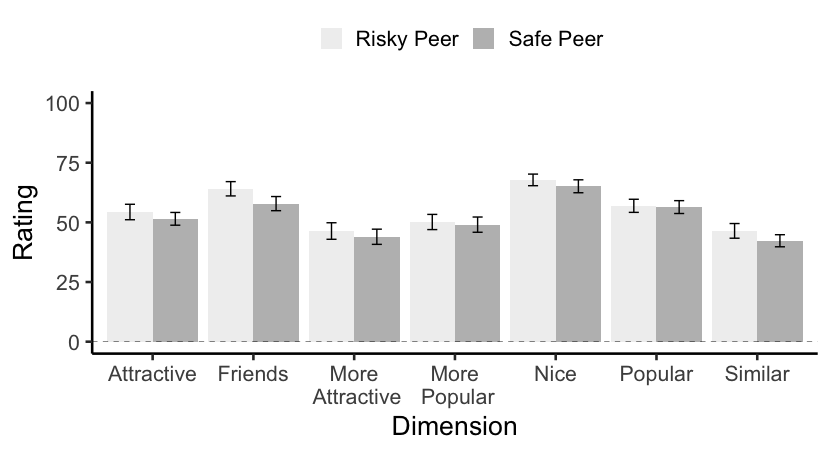


*Supplementary Figure 2.* Average ratings for the risky and safe peer. Ratings were collected before the onset of the task.


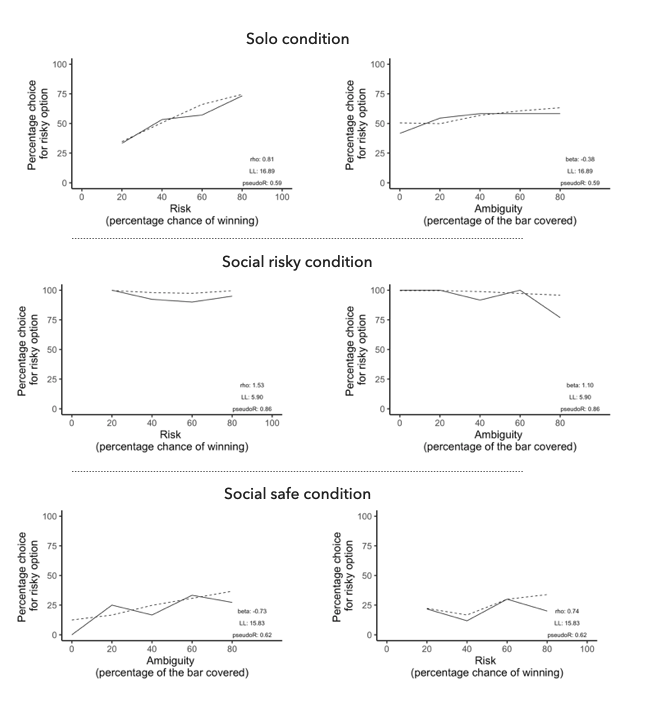


*Supplementary Figure 3.* Actual choice data (solid line) and predicted choice data (dashed line) for risk and ambiguity in all conditions for one individual participant. Plots for individual participants are available on the Open Science Framework: <https://osf.io/8frsj/>.
